# Medication deserts: survey of neighborhood disparities in availability of prescription medications

**DOI:** 10.1186/1476-072X-11-48

**Published:** 2012-11-09

**Authors:** Philippe Amstislavski, Ariel Matthews, Sarah Sheffield, Andrew R Maroko, Jeremy Weedon

**Affiliations:** 1 Department of Environmental and Occupational Health Sciences, School of Public Health, Downstate Medical Center, State University of New York, Box 43, 450 Clarkson Avenue, Brooklyn, NY 12203, USA; 2 School of Public Health, Downstate Medical Center, State University of New York, 450 Clarkson Avenue, Brooklyn, NY, 12203, USA; 3 NYCRx, 2090 Adam Clayton Powell Blvd. 5th Fl., New York, NY, 10027, USA; 4 Department of Earth Environmental and Geospatial Sciences Gillet Hall, Room 325 Lehman College, Bronx, NY, USA; 5 Scientific Computing Center, Downstate Medical Center, State University of New York, 450 Clarkson Avenue, Brooklyn, NY, 12203, USA

**Keywords:** Community pharmacy, Medication access, Medication desert, Poverty, Socio-economic status, Vehicle ownership

## Abstract

**Background:**

Only a small amount of research has focused on the relationship between socio-economic status (SES) and geographic access to prescription medications at community pharmacies in North America and Europe. To examine the relationship between a community’s socio-economic context and its residents’ geographic access to common medications in pharmacies, we hypothesized that differences are present in access to pharmacies across communities with different socio-economic environments, and in availability of commonly prescribed medications within pharmacies located in communities with different socio-economic status.

**Methods:**

We visited 408 pharmacies located in 168 socio-economically diverse communities to assess the availability of commonly prescribed medications. We collected the following information at each pharmacy visited: hours of operation, pharmacy type, in-store medication availability, and the cash price of the 13 most commonly prescribed medications. We calculated descriptive statistics for the sample and fitted a series of hierarchical linear models to test our hypothesis that the in-stock availability of medications differs by the socio-economic conditions of the community. This was accomplished by modeling medication availability in pharmacies on the socio-economic factors operating at the community level in a socio-economically devise urban area.

**Results:**

Pharmacies in poor communities had significantly higher odds of medications being out of stock, OR=1.24, 95% CI [1.02, 1.52]. There was also a significant difference in density of smaller, independent pharmacies with very limited stock and hours of operation, and larger, chain pharmacies in poor communities as compared to the middle and low-poverty communities.

**Conclusions:**

The findings suggest that geographic access to a neighborhood pharmacy, the type of pharmacy, and availability of commonly prescribed medications varies significantly across communities. In extreme cases, entire communities could be deemed “medication deserts” because geographic access to pharmacies and the availability of the most prescribed medications within them were very poor. To our knowledge, this study is first to report on the relationship between SES and geographic access to medications using small area econometric analysis techniques. Our findings should be reasonably generalizable to other urban areas in North America and Europe and suggest that more research is required to better understand the relationship of socio-economic environments and access to medications to develop strategies to achieve equitable medication access.

## Background

The community pharmacy is a critical source of medications, health services and health information to residents, [1,2] and pharmacies are especially critical in socio-economically disadvantaged communities, where access to prescription medications via online pharmacies and to health information and resources is often impaired [3]. The term “medication deserts” we introduce in this paper draws from the concept of food deserts, which is defined as low availability of nutritious food in underserved communities. Similarly, the presence of a medication desert is defined here as the low availability of the most commonly dispensed prescription medications in these areas. Little research has focused on the relationship between the SES of communities and geographic access to prescription medications at community pharmacies. The previous studies have raised concerns about the existence of systematic barriers in the timely procurement of prescription medications in disadvantaged communities. This concern is especially relevant because disadvantaged communities often have excess morbidity and mortality from chronic diseases, which require prescription medications for disease prevention and management [3-6].

The barriers to medication and pharmacy access can be differentiated into two principal groups: economic and geographic. Economic barriers may prevent individuals from procuring prescribed medication or adhering to the provider-prescribed medication regimen due to its high cost and/or lack of medication coverage.

On the other hand, the geographic location of the community of residence may affect individual’s economic or geographic access to medications. Residents that live in communities without a pharmacy or require lengthy travel to the closest pharmacy may face geographic barriers to accessing prescription medications regardless of their economic access. This is the case in rural areas of the United States (US) where the pharmacy is only accessible by car. These residents may also be at a disadvantage in accessing the range of health services and health information that the community pharmacies routinely provide.

Previous studies show that socio-economic factors, such as lack of health insurance and prescription coverage are associated with decreased access to medications, lower prescription medication use, and higher out-of-pocket spending [7,8]. In the US, among 92 million adults with chronic conditions between 2002 and 2004, over 21% were uninsured for at least 1 month during the previous year [8]. Rising medication costs worldwide are occurring alongside increases in medication utilization due to the surge in chronic diseases [9]. Several studies aimed to examine the prescription medications dispensing patterns in Europe, United States and Canada and reported significant differences and reported increases in both cost and dispensing across countries and demonstrate that differences exist in access across areas of different socio-economic status [9,10]. Higher prices and higher dispensing will likely mean greater medication expenditures for large groups of patients. For example, increased utilization of angiotensin-converting-enzyme (ACE) inhibitors, angiotensin receptor blockers (ARBs) and HMG-CoA reductase inhibitors (commonly referred to as statins) for cardiovascular disease (CVD) management will likely lead to increased costs to patients. Increasing costs associated with procurement of these critically-important medications may produce an economic barrier to access these medications by patients [11]. Increased utilization of CVD medications may also increase their cost short-term, while non-adherence, procurement and their repercussions will also likely lead to overall higher costs to the system.

In addition to the economic access to the medications spatial access needs to be considered. Differences in land use, transportation networks, population density and distribution among different regions, such as rural vs urban areas also influence spatial access to the pharmacies and thus to the medications and health information provided by them. For example, one study showed that access to Human Immunodeficiency Virus-related retroviral medications, information and related health care services differed significantly for rural and urban residents [12].

Several contextual variables in communities of residence may affect access to medications. In many communities, residents must travel, sometimes for long distances, by private or public transportation to reach a pharmacy to procure medications. Therefore, access to transportation, e.g., a private car, may impact individuals’ ability to procure medications.

This study examines primarily the quality of health services provided by the neighborhood pharmacy (proxied by the availability of the most community prescribed medications) and geographic access to the closest pharmacy (proxied by mean density of pharmacies per neighborhood). From the theoretical perspective, several approaches have been pioneered to examine the mismatch between quality of health services and the need of the population across the SES strata and spatial mismatch between the need and geographic availability of various health services and information. The Inverse Care Law posits that the availability of health resources varies inversely with need [13]. This approach exploits the notion that the market model produced stark variations in quality of health care that physicians are inversely related to the need of the populations served. “Inverse care law operates more completely where medical care is most exposed to market forces, and less so where such exposure is reduced” [13]. Some community pharmacies may not stock commonly prescribed medications. For example, a recent study demonstrated the widespread failure of pharmacies located in the poor, predominately African-American communities to stock opioid analgesics [14].

Geographic perspectives on the issue of accessibility have mostly centered around the spatial mismatch hypotheses related to employment. In New York City, this approach linked residential higher segregation level to the reduced geographical accessibility to jobs for African-Americans and other minority inner-city residents [15]. The mismatch approach to health services also related the existence of spatial disparities to the misbalance between health resources and disadvantaged population distributions [16]. A recent study linked disparities in geographic access to first-line anti-malarial medications with the poverty level of the community surrounding the pharmacy [9]. Such barriers in geographic access may impact medication procurement by the patients [2].

Even a brief review of salient literature on both topics reveals that that issues of equity loom large in the spatial distribution of health services [16-18] and contextual factors that influence health outcomes such as access to healthy foods [19-21]. Building on this earlier work, we consider the existence of pharmacy deserts. It is reasonable to assume that as with other health services, the reasons behind pharmacy siting decisions are rooted primarily in market forces and are not based on the need of the community. If we approach the problem from an equity perspective, the key question would be where is the best place to locate a pharmacy to maximize service to specific populations at highest need?

Several studies have examined the policies and economic forces thought to affect procurement of medications at the local pharmacies [7,22-25]. However, several key concerns can be raised about this earlier research. Firstly, these studies focused almost exclusively on economic access and did not include geographic access in the analysis. Yet, geographic access has already been shown to be an important factor affecting access to medications and to other health services [22,26-29]. Secondly, these studies were at the national level, and thus they did not examine the effects of socio-economic factors on geographic access at the community level. Yet, given these earlier findings about the key role the community socio-economic context plays in access to the health services, it is reasonable to hypothesize that the community’s socio-economic context may indeed have an effect on the quality and range of services that the local pharmacies provide. In particular, these local socio-economic forces may bear on the completeness of the medication inventory, and thus influence the residents’ ability to procure their prescription medications.

In addition to dispensing prescribed medications, the community pharmacies provide a range of important health information and services to the local residents [1,2]. In many areas, pharmacists also administer immunizations and deliver preventative services. For example, in the US pharmacists routinely administer influenza and pneumococcal vaccinations and some pharmacies participate in the Expanded Syringe Access Program (ESAP) providing non-prescription syringes to help prevent transmission of HIV and of other blood-borne diseases [1,30]. These activities highlight the importance of the community pharmacy as a key source of medications, health services and information. Because of this role, pharmacies are uniquely positioned to improve health outcomes in underserved communities and access to them is especially critical in disadvantaged communities, where access to other health resources is poor [3].

A number of national-level studies have examined access to medication [7,22-25]. These studies, however, focused almost exclusively on economic barriers and did not examine geographic barriers in the analysis, an important factor affecting access to medications [22,26-29]. These studies were also at the national level, and thus they did not examine the effects of socio-economic factors on geographic barriers at the community level. Earlier findings, however, suggest that a community’s socio-economic context plays a key role in access to medications. If such community differences in both types of access do indeed exist, they would suggest inequality in access to medications and to the range of other health services provided at the community pharmacy in different areas and thus should be carefully examined.

Economic conditions in the community and the geographic access to medications by the local residents may be related through a number of pathways. In particular, it is plausible to assume that the local economic conditions in the neighborhood may influence the prevalent type of pharmacies located there (small independent stores vs chain outlets), density of pharmacies and which medications are in kept in stock. The need to develop a survey methodology to examine relationships of the local socio-economic conditions on geographic access to the common prescription medications by the residents and to analyze them served as the impetus for the current study to characterize medication access at the community level.

## Methods

### Study aims

To examine the relationship between a community’s socio-economic context and the level of access to common medications and pharmacies by its residents, we hypothesized that differences are present in:

• geographic access to pharmacies across communities with different socio-economic environments, *and in*

• availability and cost of commonly prescribed medications and health services within pharmacies located in communities with different socio-economic status.

We hypothesized that a high poverty in a community may be inversely related to prescription medication access and that such effect on the quality of local pharmacy service may affect all local residents utilizing the pharmacy, regardless of their poverty status.

**Figure 1:**
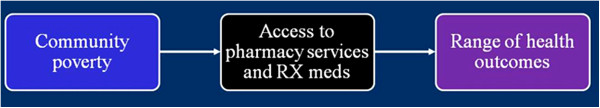
**Logic Model.** Logic model of the relationship between poverty and access to medications.

This study was approved by the State University of New York Downstate Medical Center Institutional Review Board. Our logic model describes how high community poverty negatively affects residents’ access to common prescription medications and the quality of pharmacy service they receive (see Figure 1). To better understand such an endogenic effect of the neighborhoods’ socio-economic environment on availability of medications, we considered several measures of community socio-economic status that have been shown to affect access to health services by its residents. These measures include neighborhood’s FPL, rate of private vehicle ownership, population density, and pharmacy outlet density.

### Community pharmacy mapping

We acquired a list of all active walk-in pharmacies licensed to practice in New York City from the New York State, which registers all pharmacies licensed to operate in the state.

Since we were interested in both geographic access to pharmacies and availability of commonly prescribed medications within them, we excluded pharmacies not readily accessible to the local residents, such as those located within hospitals and pharmacies that do not serve walk-in customers (e.g., pharmacies that fill prescriptions over internet only and mail-in order only). After these ineligible pharmacies were removed, we geocoded the remaining pharmacies to their street addresses. This mapping yielded a total of 2,230 pharmacies using ArcGIS 10 software (ESRI Redlands, CA). Independent pharmacies constituted 74% of all eligible pharmacies and the remaining 26% were corporately or chain-owned outlets. This represents a significant departure from the national distribution of the independent vs chain-owned pharmacies. According to the 2010 data, in the 48 contiguous US states, independently-owned pharmacies represented 49.37% of all pharmacies (Business Analyst 2010 database, ESRI, Redlands, CA). Pharmacies attached to a corporate chain store were not excluded from the sample, and when encountered, labeled as “chain” and surveyed.

### Community demographic socio-economic indicators

**Figure 2:**
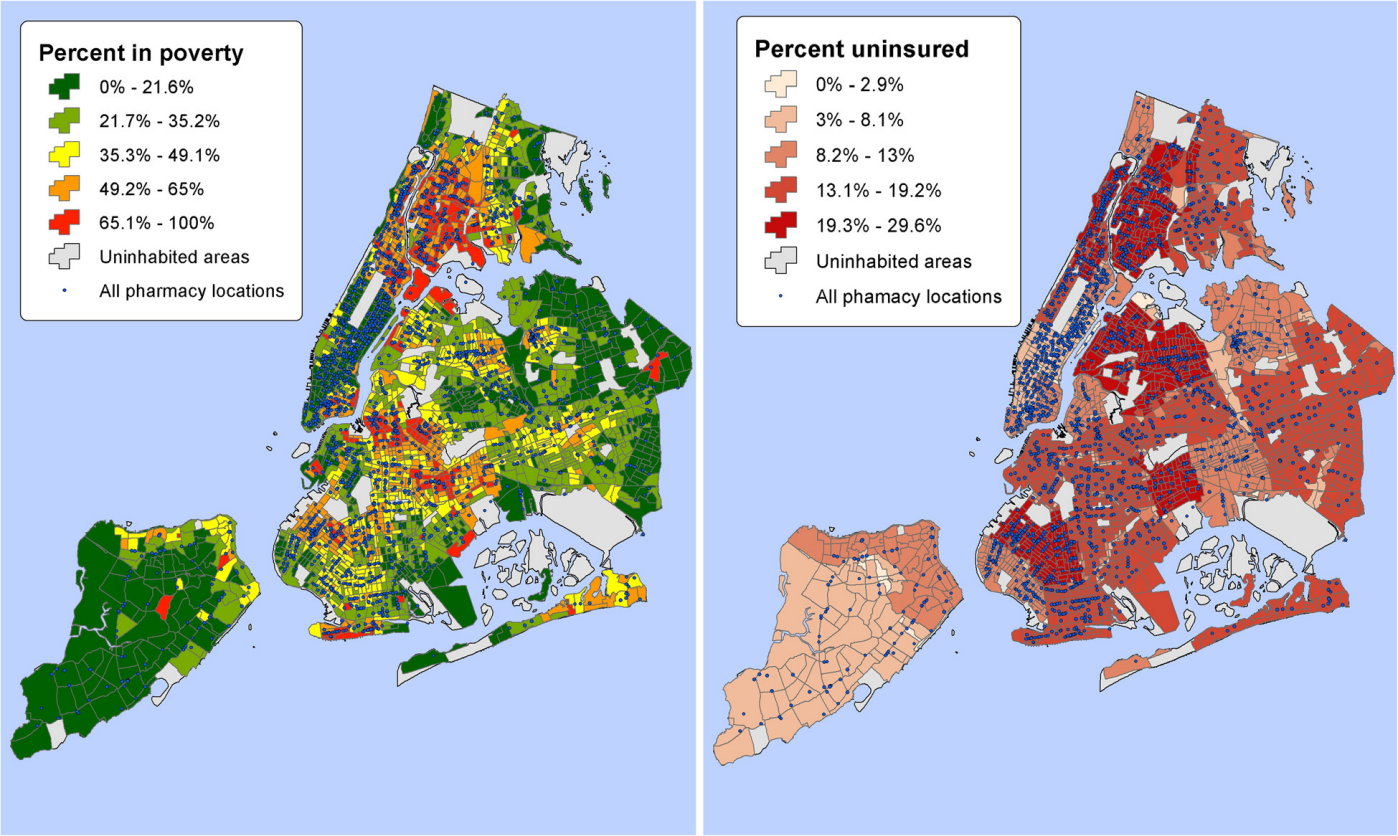
**Pharmacies and selected SES indicators by community.** Maps of all New York City pharmacies overlaid with poverty and percent of residents without health insurance.

**Figure 3:**
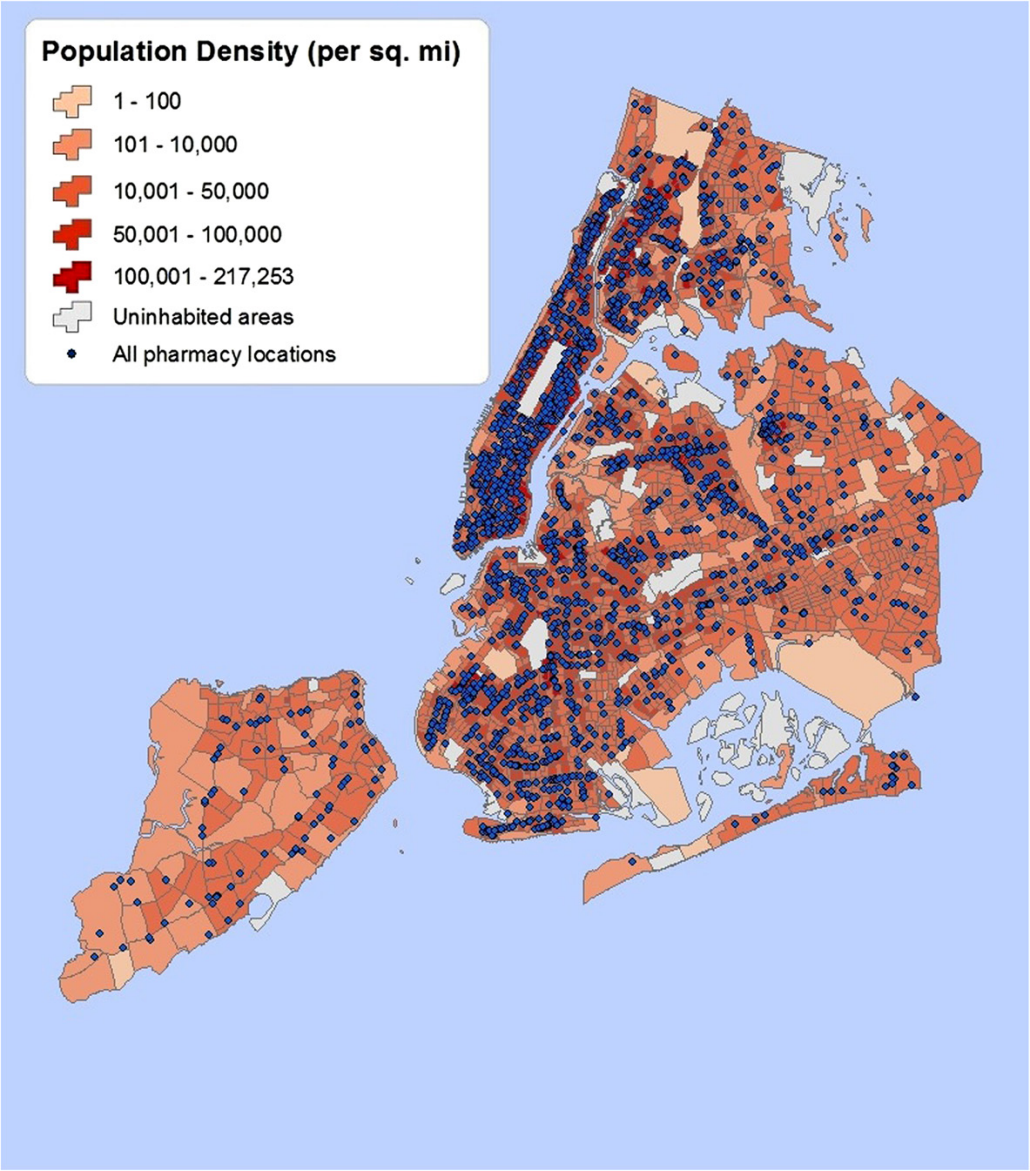
**Pharmacies and population density by community.** Map of all New York City pharmacies overlaid with population density.

The task of defining the appropriate community boundaries was important to this study. Selecting large neighborhood units would “smooth” the differences that often exist in adjacent neighborhoods and selecting small units would not accurately portray the geographic access to the pharmacies. While in the US the Census Block Group is the smallest US Census tabulation area to which aggregated socio-economic data is available, many block groups have relatively small populations, and these block groups and are small in area. Therefore, using block groups or other similarly small areal units to delimit communities would result in a smaller total community population denominator, thereby introducing larger standard error for the SES data. Thus, for the purpose of this study we opted to use a larger unit to which the SES data is aggregated, which is U.S. Census 2000 Tracts (CT) to define the community boundaries [31]. Several SES indicators which were previously shown to affect access to a range of health services were computed using the American Community Survey dataset, including the percent of population below Federal Poverty Level (FPL), defined as population with income to the Federal poverty threshold ratio below 2.00, percentage of households without a vehicle and population density per square mile in each community [32]. Additionally, percentage of residents without health insurance was computed using health insurance data from the 2011 New York City Community Health Survey (CHS). Figures 2 and 3 show the differences in geographic distribution of all pharmacies in communities overlaid with percent of residents in poverty, percentage of the uninsured, and population density. Using unique CT identifier numbers, the community-level SES indicators were later joined to the pharmacy survey data for sampled communities to test various hypotheses about relationship between the community’s socio-economic context and geographic access to prescription medications by its residents using regression models.

### Survey instrument development and sample selection

**Figure 4:**
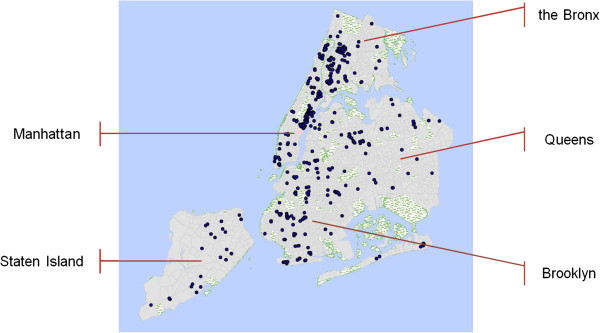
**Surveyed pharmacies.** Map of surveyed pharmacies located within the sampled communities.

We designed a survey tool to assess both the availability of prescription medications and the provision of pharmacy services. The City of New York is comprised of five boroughs, namely Brooklyn, the Bronx, Manhattan, Queens and Staten Island (Figure 4). Each of these large administrative units is distinctive infrastructurally, demographically, socio-economically and culturally. Pharmacy density and population distributions also vary widely among the boroughs, with Manhattan having the highest number of pharmacies per capita. We created a database of all communities (defined as individual CTs) that had at least one pharmacy present and then randomly sampled 5% of communities with high, medium, and low poverty rate respectively, in all boroughs of New York City except Manhattan. Since Manhattan had only 39 CTs in the middle poverty group with pharmacies, we modified our selection schema for that borough by oversampling Manhattan CTs at the rate of 20%. Random selection at that rate produced 8 tracts in the “middle poverty” group in Manhattan. This sampling method yielded a sample of 408 pharmacies in 168 communities in all five boroughs of New York City.

The survey collected the following information: hours of operation, pharmacy type (chain or independent), and the quantity, cash price, and current availability of the 13 most prescribed medications. The 13 prescription medications included the top 6 brand and 7 generic medications by total prescriptions written [33]. The products for which we collected availability and pricing data and the conditions for which they are prescribed are listed in the Table 1.

**Table 1:** **Surveyed medications**, **their indications**, **mean price**/**tablet for each medication and its standard deviation**, **minimum and maximum prices in the survey sample**

**Medication name**	**Indications**	**Mean price per tablet**	**SD**	**Minimum price**	**Maximum price**
Brand medications					
Lipitor® (simvastatin calcium)	High cholesterol	4.32	0.7	2.1	6.37
Nexium® (esomeprazole magnesium)	Acid reflux, gastroesophageal reflux disease (GERD)	7.14	0.91	4.2	13.1
Lexapro® (escitalopram oxalate)	Depression	4.28	0.51	2.39	6.93
Singulair® (montelukast sodium)	Asthma	5.63	0.85	1.93	8.23
Actos® (pioglitazone HCl)	Diabetes	9.4	1.15	5.17	13.07
Plavix® (clopidogrel bisulfate)	Stroke and heart attack prevention	7.29	1.12	3.77	15
Generic medications					
^1^Hydrocodone-APAP	Pain relief	0.62	0.3	0.16	2.67
Lisinopril	High blood pressure	0.89	0.36	0.13	1.94
Simvastatin	Diabetes	2.35	1.65	0.14	8.37
Levotheroxine	Hypothyroidism	0.6	0.31	0.13	2.84
Amoxicillin	Bacterial infections	0.61	0.23	0.13	3.16
Metformin	Diabetes	0.86	0.62	0.13	2.81
Hydrochlorothiazide	Hypertension	0.36	0.14	0.13	1.2

### Survey administration

Pharmacy and public health students were trained in survey administration. The interviewers visited all pharmacies in the selected 168 communities (See Figure 4).

Surveys were conducted during the daylight hours, during weekdays, and at various times during the day according to the schedules of the interviewers. Upon entry to the store the interviewers introduced themselves, showed student identification and explained the purpose of the visit to the pharmacy staff using a standard script. The survey-takers asked to speak with the pharmacist or, if the pharmacist was not available, a pharmacist technician. The students asked to view a copy of the pharmacy’s retail drug pricing list. If the pharmacy declined to participate, the students thanked them for their time and left the location promptly, taking note for the pharmacy to be flagged as a “refusal” during data entry. We only collected data on the pharmacies who agreed to participate and provided the requested information at the time of the visit. The pharmacy participation rate in the survey was 86.3% (n=352) out of the 408 pharmacies identified and visited. If the pharmacy agreed to provide pricing information for the prescription drugs, the students used the pricing list to fill in the survey instrument, and then searched the shelves for OTC items to obtain those prices (not reported here). Using the survey form, the interviewers assessed the availability and cost of each of the medications on the survey form at the time of the visit by interviewing the pharmacist on staff and entered the pharmacist’s responses into the spreadsheet.

Completed surveys were entered by survey-takers. The completed database contained pharmacy address information, its community’s CT number, coding for income level, type, hours of operation (whether the outlet is open Saturday, Sunday, or offers 24 hour service), price per container of each item, amount of tablets per container, and price per pill information for all 25 products, and counts of how many items were out of stock. Out of stock counts were used to create the study’s dependent variable, defined as the probability of having one or more prescription items out of stock.

After this pricing data file was complete, one additional data file was created. This file provided descriptive socio-economic data on the sampled communities, such as chain and independent pharmacy density per 1,000 residents, percent households without car, percent non-Hispanic black residents, % Hispanic residents, and median annual household income [32]. Data on these variables for the surveyed communities in Manhattan was reported separately (Table 2). A subset of these census tract level variables was used in regression analysis as independent variables describing the neighborhoods’ socioeconomic status to model their hypothesized relationship with the medication access at the local pharmacies.

**Table 2:** Communities characteristics and access to pharmacy by community poverty level

**Community characteristics**	**Community federal poverty level(range)**			
	**High poverty**	**Medium poverty**	**Low poverty**	**ANOVA result**
Total number of surveyed communities in outer boroughs(n)	**43**	**67**	**17**	
Total pharmacy density per 1,000 residents outer Boroughs (mean ± SD)	0.51 (± 0.35)	0.62 (± 0.51)	0.35(± 0.17)	**p**=**0**.**021**^**1**^
Density of independent pharmacies per 1,000 residents except Manhattan (mean ± SD)	0.39 (± 0.30)	0.40 (± 0.39)	0.13 (± 0.21)	**0**.**015**^**2**^
Density of chain pharmacies per 1,000 residents except Manhattan (mean ± SD)	0.06 (± 0.13)	0.22 (± 0.19)	0.17 (± 0.19)	**p**=**0**.**002**^**2**^
% Households without car (mean ± SD)	75.57 (± 8.02)	51.22 (± 20.17)	30.35 (± 22.55)	**p**<**0**.**001**^**2**^
% Non-Hispanic Black residents (mean ± SD)	28.98 (± 21.97)	19.09 (± 0.17)	14.76 (± 29.13)	p=0.06
% Hispanic residents (mean ± SD)	53.91 (± 22.77)	27.61 (± 23.93)	8.35(± 4.81)	**p**<**0**.**001**^**2**^
Median Annual Household Income (in US Dollars)	21,419(± 6352)	45,768 (± 16008)	85,177 (± 16265)	**p**<**0**.**001**
Total number of surveyed communities in Manhattan (n)	**10**	**7**	**23**	
Total pharmacy density per 1,000 residents in Manhattan (mean ± SD)	0.68 (± 0.37)	0.24 (± 0.10)	0.51 (± 0.38)	**p**=**0**.**002**^**1**^
Density of independent pharmacies per 1,000 residents in Manhattan (mean ± SD)	0.43 (± 0.3)	0.21 (± 0.10)	0.10 (± 0.14)	**p**=**0**.**007**^**1**^
Density of chain pharmacies per 1,000 residents in Manhattan (mean ± SD)	0.25 (± 0.29)	0.01 (± 0.04)	0.38 (± 0.39)	**p**<.**001**^**4**^
% Households without car (mean ± SD)	77.84 (± 6.15)	78.35 (± 5.05)	72.87 (± 7.35)	p=0.068
% Non-Hispanic Black residents (mean ± SD)	32.00 (± 26.95)	34.29 (± 29.81)	1.57 (± 1.16)	**p**=**0**.**005**^**3**^
% Hispanic residents (mean ± SD)	51.80 (± 31.62)	37.14 (± 20.65)	4.78 (± 1.57)	**p**<**0**.**001**^**1**^
Median Annual Household Income in Manhattan (in US Dollars)	30,255 (± 11645)	42,182 (± 20408)	11,8358 (± 28982)	**p**<**0**.**001**^**1**^

^1^Pairwise comparisons among the means of Communities with all three levels of poverty statistically significant.

^2^ANOVA was statistically significant and so was pairwise comparison of High vs Medium Poverty Communities.

^3^ANOVA was statistically significant and so was pairwise comparison of Low vs Medium Poverty Communities.

^4^ANOVA was statistically significant and so was pairwise comparison of High vs Low Poverty Communities.

The CT-level estimates from the American Community Survey 2005–2009 data were used to create the study’s independent variables describing the neighborhood socioeconomic status. One limitation of the American Community Survey data is that it is based on estimates derived from a sample of the CT's population, which can introduce large errors when an estimate is calculated for CTs with small populations. However, this is the best SES data publicly available in the United States and in effort to reduce error associated with this issue we excluded pharmacies with CTs of less than 100 persons from our survey. The aggregated data on health insurance status in New York City is not available on the Census Tract level. Therefore, we used insurance data from the New York City Department of Health and Mental Hygiene data from the annual Community Health Survey (CHS) and apportioned it to the census tracts to utilize in the analysis.

### Statistical analysis

After basic descriptive statistics for the sample were calculated, we fitted a series of multilevel models to test our hypothesis that the in-stock availability of the most commonly-prescribed medications differs by the socio-economic conditions of the community. All statistical analyses were performed with SAS 9.2 software. The key independent variable of interest was the FPL of the community. Covariates for this analysis included percentage of households without a vehicle, community population density per unit of area, pharmacy density per unit of population, and percentage of residents without health insurance. Total pharmacy density, density of the independent pharmacies and of the chain pharmacies were computed per 1,000 residents. Car ownership was computed as a percentage of the households without cars. We also determined the percentage of the Non-Hispanic Black residents, Hispanic residents, and the median annual household income in each community. As a proxy of geographic access to any pharmacy in the neighborhood, we computed pharmacy density per 1,000 residents in each community by dividing total population by the total number of pharmacies geocoded in this community. We used the density of total number of neighborhood pharmacies per 1,000 residents, and density of the independent pharmacies and of the chain pharmacies per 1,000 residents as proxies of geographic access to any pharmacy, to the independent pharmacies and to the chain outlets, respectively. We separated independent from the chain pharmacies because of their inherent differences in the type and scope of services provided.

Several kinds of generalized mixed linear models were fitted: Poisson; zero-inflated Poisson; binomial; negative binomial; and Bernoulli with categories 0 versus > 0 (since 85% of pharmacies had score zero). Models were compared using the Akaike information criterion.

Polynomials in the predictors were added as necessary to achieve adequate response functions. The presence of a random effect associated with community was tested. Likelihood ratio (LR) methods for estimation of p-values and confidence intervals (CI) were used. Model residuals were checked for outliers.

## Results

The dependent variable for geographic accessibility of prescription medications within the community of residence was the probability of any of the top 13 prescription medications in the community pharmacy being out of stock or otherwise unavailable at the time of the survey. 85% of the surveyed pharmacies had all 13 prescription medications available. Of the 15% of pharmacies that had at least one item out of stock, one item was missing from 36% of these pharmacies with out-of stock prescription items. 74.5% of these pharmacies had five or fewer items missing. The remaining 25.5% had six or more items missing. The most common occurrence in this category was having six items missing, followed by all 13 missing. There appeared to be some spatial clustering of pharmacies with prescriptions out of stock in Manhattan and in the Bronx, but the number of pharmacies involved in our sample was too small to ascribe any statistical significance to this using a cluster search tool (SatScan, v9.1).

Table 1 provides a list of the 13 surveyed medications, their indications, mean cash prices per tablet for each medication and its standard deviation, minimum and maximum prices in the survey sample.

Table 2 describes the selected socio-economic and demographic characteristics of the sampled communities in the five New York City boroughs by the poverty level, defined as high, medium and low poverty based on the percentage of households below the 200 percent of Federal Poverty Level. The data were compared using ANOVA test; p < 0.05 was considered a statistically significant difference between the communities surveyed. The ANOVA p-values highlighted in bold are significant at p > 0.05 level. Manhattan has very different geographic, socio-economic and demographic characteristics compared to the other four New York City Boroughs. Therefore, we present the descriptive data on Manhattan separately from the other boroughs.

From Table 2 independent pharmacies are more likely to be found in the poorer neighborhoods of New York City (p=0.021) while chain pharmacies are more likely to be located in lower poverty areas (p=0.01). It is noteworthy to mention that in some cases the overall ANOVA result for differences in community characteristics and access to pharmacy (proxied by pharmacy density) by the community poverty level was statistically significant, while the pairwise comparisons among the means were not (please refer to the table legend). This is plausibly due to the small sample size in some of the groups.

Pharmacies open 24 hours were 5% of the respondents (n=17), these open on Saturday were 91% (n=322), open on Sunday were 47% (n=166), open both Saturday and Sunday were 45% (n=159) and closed both Saturday and Sunday were 7% (n=23).

We were primarily interested in the medications out of stock in the pharmacies surveyed (Table 3). Over 85.5% of pharmacies reported all the medications surveyed as being in stock. Among the 51 pharmacies reporting missing medications the most frequent case was just one medication out o f stock (16 pharmacies), but the next most frequent case was 5 medications out of stock.

**Table 3:** Frequencies of pharmacies with the 13 most commonly prescribed medications being out of stock

**Count of unavailable and RX**	**Frequency**	**Percent**	**Cumulative frequency**	**Cumulative percent**
0	301	85.51	301	85.51
1	16	4.55	317	90.06
2	5	1.42	322	91.48
3	3	0.85	325	92.33
4	2	0.57	327	92.9
5	11	3.13	338	96.02
6	7	1.99	345	98.01
7	1	0.28	346	98.3
10	1	0.28	347	98.58
13	***5***	***1***.***42***	***352***	***100%***

### Regression analysis of survey data

The best-fitting regression model was that associated with the Bernoulli distribution, with the outcome dichotomized as zero vs more than zero. The CT random effect was of marginal statistical significance (p=0.085), but the presence or absence of this effect in the model made very little difference to either estimates or p-values of the fixed effects; for simplicity, results reported here do not include the effect. A model containing linear and quadratic terms in % of households without cars, plus linear terms in the other predictors satisfied the Hosmer-Lemeshow lack of fit test (χ^2^_8_=2.08, p=0.978).

From Table 4, adjusted odds ratio (OR) for a 10 percentage-point increase in population below 200% of Federal poverty income level for all NYC boroughs was 1.24, with 95% CI [1.02, 1.52]. In other words, for each 10 percentage-point increase in the number of households in poverty, odds of one or more prescription medications unavailability on the pharmacy shelves increases by 24 %, and this increase had a roughly uniform character across the range of the data.

**Table 4:** **Results of multiple predictor generalized linear model** (**GLM**) **for all boroughs of New York City**

**Model parameter**	**Odds ratio for 10%point increase in odds of being out of stock**	**95%CI for 10%point increase**		**χ2(df=1)**	**p-value**
% uninsured population	0.8496	0.4579	1.5516	0.28	0.597
% residents in poverty	**1**.**24**	1.02	1.52	4.47	**0**.**035**
% households without a car	**0**.**414**	0.2001	0.8668	5.42	**0**.**020**
% households without a car squared	1.01	0.99	1.0141	3.26	0.071

Car ownership also was shown to have statistically significant effect in the opposite direction. Each percentage-point increase in the number of the number of households with a private car in the community was associated with increased odds of availability of most common prescription medications in the local pharmacy.

A LR test of the null hypothesis that both car ownership terms are zero yielded χ^2^_2_=7.29, p=0.026. ORs for a 1 percentage-point increase in % of households with no car [95% CI] (estimated at minimum, maximum and quartile values) were 0.923 [0.866, 0.985] (at min=6.3%), 0.989 [0.964, 1.016] (at Q1=58.3%), 1.005 [0.968, 1.043] (at Q2=69.8%), 1.017 [0.969, 1.067] (at Q3=78.9%), and 1.035 [0.969, 1.106] (at max=92.5%). Put differently, the effect of vehicle occurs only in tracts with high vehicle availability; among such tracts, greater vehicle unavailability is somewhat associated with reduced prescription medication unavailability.

Analysis of pharmacies in Manhattan only (N=110) showed the same general pattern with the same set of significant predictors (% households without a car had p=0.013). The OR for % poverty was 1.077, with 95% CI [1.029, 1.137].

## Discussion

The findings of this study demonstrate that geographic access to a neighborhood pharmacy and the type of pharmacy (independent versus chain outlet), and to medications within pharmacies varies significantly across the communities in a large and socio-economically diverse urban area. These findings suggest that a similarly wide variation in access may exist in urban areas of other economically developed countries.

While only 14.5% of pharmacies in the sample reported missing medications and most of these outlets had multiple medications out of stock (Table 3). This finding suggests that in some neighborhoods patients with several conditions for these medications prescribed would be able to fill their prescriptions upon the visit to the pharmacy. This situation could adversely affect patients with multiple co-morbidities.

**Figure 5:**
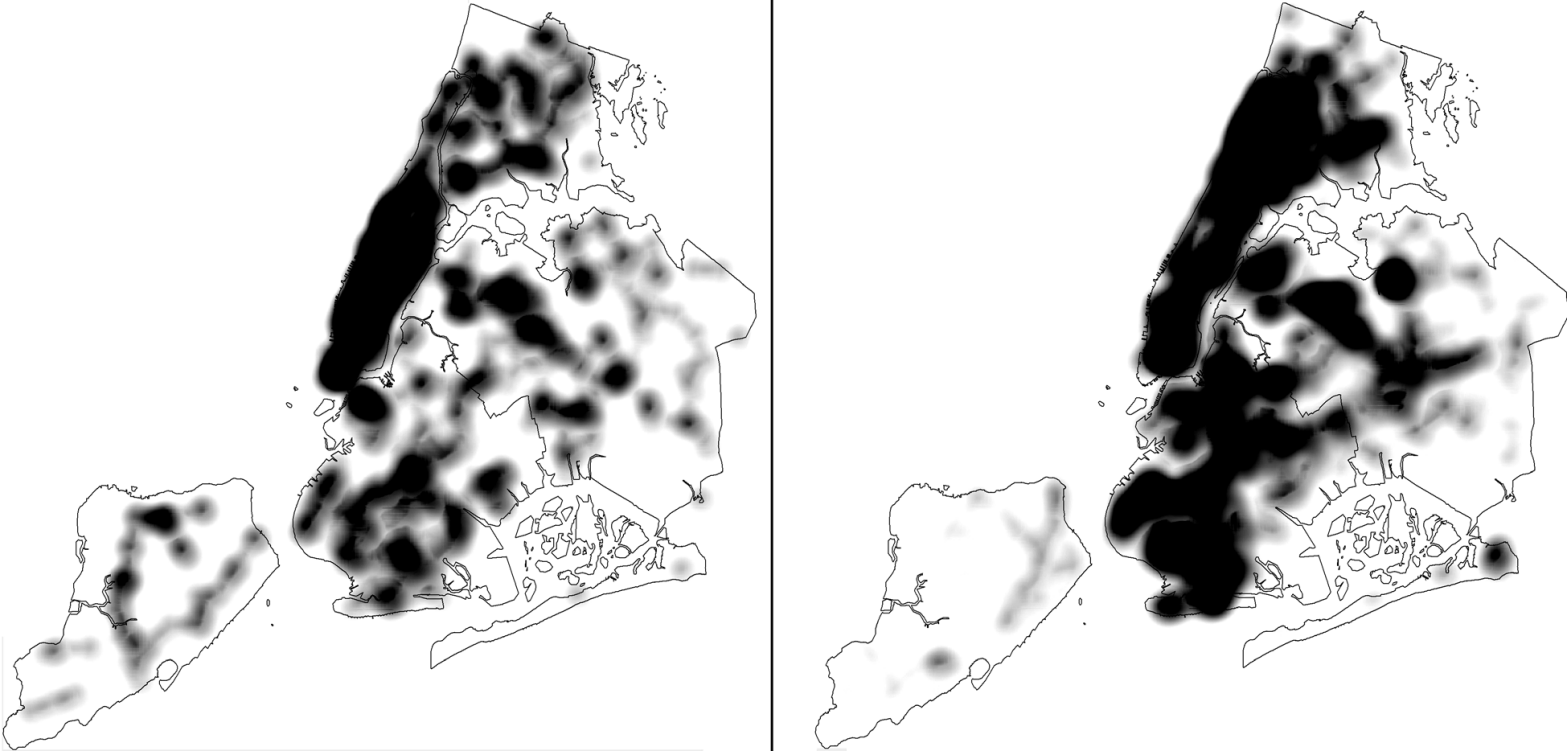
**Kernel density estimates.** Kernel density estimates (KDE) of ½ mile bandwidth for chain pharmacies (left image) versus independent pharmacies (right image).

As Table 4 shows, there is a significant difference in density of chain pharmacies in the poorest communities as compared to the middle- and low-poverty communities in the outer boroughs. This observation is important for future initiatives focused on disease prevention and management. This is because of the different services independent and chain pharmacies are often able to provide to the neighborhood. Chain pharmacies typically have larger facilities and thus may have a wider range of prescription medications in stock than independent pharmacies and may be able offer a somewhat broader range of preventive health services, including vaccinations administered by the pharmacists and self-service blood pressure monitoring. They are also more likely to remain open and to have a pharmacist in the store to disperse prescription medication at night, on the weekends and holidays. Yet, residents of the poorest communities in the “outer” boroughs of New York City (Brooklyn, Bronx, Queens, and Staten Island) have the least geographic access to chain pharmacies at only 0.06 (± 0.13) pharmacies per 1,000 residents, as compared to the 0.22 (± 0.19) pharmacies per 1,000 residents in the middle poverty communities. The visual comparison of the ½ mile bandwidth kernel density estimates (KDE) for chain pharmacies versus independent pharmacies reveal significant geographic differences in both geographic distribution and density of the chain pharmacies in poor and in outlying parts of the city (see Figure 5).

Fitting the generalized linear models to our data demonstrated that higher community poverty and lower access to a private vehicle have a statistically significant association with unavailability of the most commonly prescribed medications. This may have serious consequences in access to the prescribed medications and a whole range of other health services by the local residents.

The findings are especially important as poor communities have the highest prevalence and incidence of chronic illnesses for which medications are commonly prescribed [6] and our results suggest that patients in poor areas, where there is often a higher need for these medications due to higher disease burden, may experience greater difficulty in filling their prescriptions.

A potential consequence of disparity in medication availability in poor areas is that residents have to travel farther outside of their communities to more affluent areas with pharmacies stocking their medications. However, many poor residents are unable, due to lack of private car and of convenient public transportation, to travel outside their communities to procure these prescription medications. Even if the local pharmacy is willing to order the out-of-stock medication, the patient will need to postpone taking it until the needed medication is delivered. In all of the above scenarios, the care of the patients living in such “medication deserts” is negatively affected. While this study did not assess the effect of the economic and geographic barriers to accessing medications on medication compliance, it is reasonable to hypothesize that these barriers directly or indirectly affect medication compliance and this question needs to be explored by the future research.

One explanation of the higher odds of common medications being out-of stock in poorer communities is that in areas with high poverty and low rates of prescription insurance coverage, pharmacy operators may decide not to stock some high-cost prescription medications because they may not be able to sell them. This explanation has also been offered by some of pharmacy operators located in low-income communities with high percentage of residents without health insurance during our study, who disclosed that they often charge higher prices for medications purchased with cash by patients without health insurance and without prescription coverage. Clearly, such pricing practices could adversely affect access to prescription medications for patients without prescription coverage in low-income area with large percentage of residents without prescription coverage and need to be examined in greater depth.

Another question that needs to be examined in the future research is whether poor geographic access to medications is associated with reduced prescription medication compliance in local residents because of the difficulties procuring it. Published research demonstrates that high-poverty is linked to higher prevalence and incidence of the very conditions that the medications we surveyed are used to treat: asthma, stroke, type 2 diabetes, hypercholesterolemia, hypertension and heart disease [1]. It would be relevant to further explore the nature of the relationship between availability of specific groups of medications in the community pharmacies and the health outcomes that these medications are used to treat.

### Limitations

We used “lack of health insurance” as a proxy to lack of prescription medication coverage. It is possible that prescription coverage is somewhat independent of health insurance status and that these benefits would vary among countries.

Since medications available would vary according to restocking periods for each pharmacy, and information on restocking was unavailable to the study team, this is inevitably a limitation of this study. However, as pharmacies were surveyed on different weekdays and at different times this limitation should not affect generalizability of our findings. Other potential sources of error were due to ambiguous labels, errors in correctly recognizing medication names and quantity of medication units in packages or containers of different sizes and data entry errors. An additional limitation of this study is our use of the number of pharmacies per person in a CT as a proxy of geographic access to the medications. A more robust measure of geographic access, such as kernel density estimates, may be a better approach and will be incorporated into the future steps of this research.

Since this study was focused on access to medications in the community as experienced by its residents, we did not consider other avenues of procuring prescription medications, such as via the Internet, at a mail-in pharmacy or through a health care provider’s office or clinic. Another limitation of this study is lack of data on medication procurement at the patient level and therefore the individual procurement-related behaviors are unknown. Due to ecological nature of this study, we did not examine access to the mediations outside one’s CT of residence. Specifically, our somewhat restrictive accessibility focus, defined by the CT of residence, has a limitation in that it does not examine the effect of procurement of prescription medications outside of their communities on the total medication access. For example, we did not study how individuals might draw on pharmacies closer to other anchor locations in their daily life (near work, along transportation routes) or procurement by a family member or social network member.

It is important to understand patient procurement-related behavior to better leverage the pharmacies’ potential to improve future disease prevention and management. A better understanding of procurement behavior could bring a much-needed component to this initial look into the interplay between community poverty and medication access.

To the best of our knowledge, this study is the first to report on disparities in access to and availability of common prescription medications in a large ethnically and socio-economically diverse urban areas and our methods should be reasonably generalizable to other large population centers in the developed countries.

## Conclusions

To the best of our knowledge, this study is the first to report on disparities in access to and availability of common prescription medications in a large ethnically and socio-economically diverse urban areas and our methods should be reasonably generalizable to other large population centers in the developed countries.

These initial findings suggest that geographic access to a neighborhood pharmacy, the type of pharmacy, and in-stock availability of the most commonly prescribed medications within the pharmacy varies significantly across communities. In extreme cases, entire communities could be deemed “medication deserts” because geographic access to pharmacies and the availability of the most prescribed medications within them were very poor. More research is required to better understand the relationship of socio-economic environments and geographic access to most common prescription medications to develop effective strategies to achieve equitable access to prescription medications in disadvantaged communities.

## Competing interests

The authors declare that they have no competing interests.

## Authors’ contributions

PA conceived of the study, designed it, contributed to the analysis and drafted the manuscript. AM participated in the survey design, carried out the survey administration and managed the study database. SS participated in survey development and guided the student interns. ARM participated in study design and helped with the spatial apportionment of health insurance data. JW participated in the design of the study and performed the statistical analyses. All authors read and approved the final manuscript.
